# The Effect of Blue Light on the Production of Citrinin in *Monascus purpureus* M9 by Regulating the *mraox* Gene through lncRNA *AOANCR*

**DOI:** 10.3390/toxins11090536

**Published:** 2019-09-13

**Authors:** Hua Yang, Xufeng Wang, Zhenjing Li, Qingbin Guo, Mingguan Yang, Di Chen, Changlu Wang

**Affiliations:** 1 State Key Laboratory of Food Nutrition and Safety, Key Laboratory of Food Nutrition and Safety, Ministry of Education, College of Food Engineering and Biotechnology, Tianjin University of Science and Technology, Tianjin 300457, China; yanghua181@tust.edu.cn (H.Y.); wangxufeng@mail.tust.edu.cn (X.W.); lizhenjing@tust.edu.cn (Z.L.); guoqingbin008322@tust.edu.cn (Q.G.); mgyang@qlu.edu.cn (M.Y.); 2 College of Biological Engineering, Henan University of Technology, Zhengzhou 450001, China; chendi1906@haut.edu.cn

**Keywords:** *Monascus*, citrinin, lncRNA, blue light, nephrotoxicity

## Abstract

Blue light, as an important environmental factor, can regulate the production of various secondary metabolites of *Monascus purpureus* M9, including mycotoxin-citrinin, pigments, and monacolin K. The analysis of citrinin in *Monascus* M9 exposed to blue light for 0 min./d, 15 min./d, and 60 min./d showed that 15 min./d of blue light illumination could significantly increase citrinin production, while 60 min./d of blue light illumination decreased citrinin production. Analysis of long non-coding RNA (LncRNA) was performed on the transcripts of *Monascus* M9 under three culture conditions, and this analysis identified an lncRNA named *AOANCR* that can negatively regulate the *mraox* gene. Fermentation studies suggested that alternate respiratory pathways could be among the pathways that are involved in the regulation of the synthesis of citrinin by environmental factors. Aminophylline and citric acid were added to the culture medium to simulate the process of generating cyclic adenosine monophosphate (cAMP) in cells under illumination conditions. The results of the fermentation showed that aminophylline and citric acid could increase the expression of the *mraox* gene, decrease the expression of lncRNA *AOANCR*, and reduce the yield of citrinin. This result also indicates a reverse regulation relationship between lncRNA *AOANCR* and the *mraox* gene. A blue light signal might regulate the *mraox* gene at least partially through lncRNA *AOANCR*, thereby regulating citrinin production. Citrinin has severe nephrotoxicity in mammals, and it is important to control the residual amout of citrinin in red yeast products during fermentation. LncRNA *AOANCR* and *mraox* can potentially be used as new targets for the control of citrinin production.

## 1. Introduction

*Monascus* is a traditional Chinese medicinal and food homologous fungus. Its secondary metabolites mainly include pigments, monacolin K, and citrinin. Monacolin K is the main lipid-lowering functional substance of *Monascus* and it has a competitive inhibitory effect on HMG-CoA reductase and inhibits cholesterol synthesis [1]. *Monascus* pigments are a mixture of compounds with a similar structure (azone-based compounds), which is a natural pigment widely used in food coloring. There are three categories: yellow pigments, orange pigments, and red pigments [2,3,4]. They have many physiological activities, such as cytotoxicity, immunosuppressive activity, anti-inflammatory activity, and improving metabolic syndrome [5,6,7,8,9]. Citrinin is a mycotoxin with nephrotoxicity, genotoxicity, carcinogenicity, embryotoxicity, and teratogenicity in mammals [10]. Since the discovery of citrinin in 1979, its toxicity has received increasing attention.

There are two main sources of citrinin residues: (1) *Monascus*-contaminated soybeans, sorghum, rice, oats, and related products [11]; (2) citrinin is often contaminated in red yeast products that contain pigments, since the citrinin and pigments have a concomitant relationship. The safety of *Monascus* products has attracted worldwide attention due to the existence of citrinin [12,13].

In addition to red yeast products, other products that are susceptible to mycotoxin contamination, such as food, animal feed, etc., have also been found to contain citrinin. [14]. A report on mycotoxin exposure in Portugal showed that citrinin can be detected from urine samples [15]. Other studies found that citrinin can synergize with other mycotoxins, such as ochratoxin A, thereby increasing the toxicity of each compound [16]. Countries and regions have set standards for the level of citrinin residues in red yeast products. For example, the Japanese standard limit is 0.2 μg/g and the European Union’s (EU’s) standard limit is 2 μg/g [17].

Previous researchers used biodegradation methods to reduce the amount of citrinin residues in red yeast products, such as the addition of the citrinin-degrading bacteria *Cryptococcus podzolicus* to red yeast products and the reduction of the amount of citrinin residue by heat treatment with *Saccharomyces cerevisiae* [18,19]. These methods are capable of degrading citrinin in large amounts, however they are difficult to carry out in the production process.

Citrinin contamination in red yeast products is produced simultaneously with the other useful secondary metabolites (pigment and monacolin K) during the fermentation of *Monascus* [20]. Therefore, controlling the synthesis of citrinin during the fermentation phase is the most fundamental method for reducing citrinin contamination.

Blue light can affect mycelial growth and promote the sporulation of *Monascus* [21,22], regulate the production of citrinin, monacolin K, and pigment, and regulate pigment composition [4,23,24] from the level of gene expression. Blue light regulates the expression of more than 1000 genes of *Monascus*, including ones involved in the metabolism of carbon, nitrogen, aromatic amino acids, fatty acid, etc.

Long non-coding RNA (lncRNA) is a kind of non-coding RNA [25] that is longer than 200 nucleotides and it does not have the function of coding protein. LncRNA plays an important role in cell differentiation, metabolites, and other biological processes.

Light can regulate the physiological activities of organisms by activating some lncRNA of microorganisms. A total of 939 new types of lncRNA have been isolated from *Neurospora crassa*, 11 of which can be up-regulated by light stimulation [26]. Thirty-six lncRNAs of *Chlamydomonas reinhardtii* are photosensitive [27]. However, studies of the light-induced regulation of lncRNA in microorganisms are still at an initial stage. It remains unknown whether lncRNA is involved in the regulatory effect of blue light on the secondary metabolites of *Monascus*. In this study, bioinformatic analysis of lncRNA was carried out based on the transcriptome data of the study of Di [28]. A total of 12455 RNA fragments were found to be consistent with lncRNA characteristics. Among them, 49 putative lncRNAs were sensitive to blue light induction different (false discovery rate (FDR) ≤ 0.05, fold change ≥ 2). After screening, an lncRNA, named *AOANCR*, was identified that can trans-regulate the gene *mraox*, which encodes alternate oxidase. This lncRNA might be a new regulator of the citrinin synthesis in *Monascus*.

The alternate oxidase pathway is an important pathway for *Monascus* to sense external environmental signals, especially environmental stress. The gene *mraox*, which encodes alternate oxidase, is one of the key genes of the alternate oxidase pathway that can enhance the resistance of *Monascus* and promote spore germination under environmental stress [29]. In this study, we added salicylhydroxamic acid (alternative respiratory pathway inhibitor) to the culture medium; it was found that the yield of citrinin increased, which indicated that the alternative respiratory pathway also regulates the production of citrinin. Based on the results of this study, we speculate that blue light regulates *mraox* to affect the production of citrinin by regulating lncRNA *AOANCR*.

## 2. Results

### 2.1. Effect of Blue Light on Citrinin Production in M9

**Figure 1:**
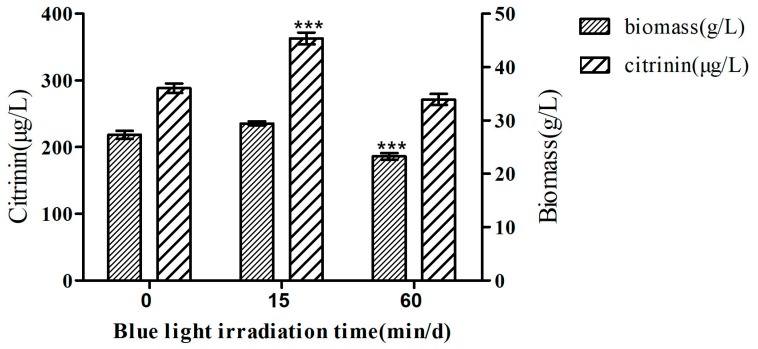
The effects of blue light on citrinin production and biomass. Blue light was generated by a blue-LED box which provided stable and uniform pure blue light (460 nm). The light intensity was 100 lux. The samples were treated with blue light once a day, for eight days of culturing. Citrinin was detected by high performance liquid chromatography (HPLC), and the biomass is expressed as the dry weight of mycelium per unit fermentation volume. The data represent the mean ± SD of six replicates. *** *p* < 0.001 compared with biomass and citrinin production for 0 min./d blue light illumination. T-test was performed at *p* < 0.05.

A culture medium inoculated with *Monascus* M9 seed solution was placed under blue light with a light intensity of 100 lux for 0, 15, and 60 min. on one day, with six solutions that were placed in parallel in each group. After eight days of culturing, the yield of citrinin and biomass (dry weight) in fermentation products were determined, as shown in Figure 1.

The biomass of *Monascus* M9 increased from 27.3 g/L in the dark to 29.5 g/L in blue light (15 min/d), however the difference was not significant. The biomass of *Monascus* M9 significantly decreased from 27.3 g/L in the dark to 23.27 g/L in blue light (60 min/d). This might be due to the fact that, although blue light is harmful to the growth of *Monascus* M9, the short illumination time may stimulate the compensatory growth of *Monascus*.

The citrinin yield of *Monascus* M9 cultured in blue light (15 min/d; 363 μg/g) was significantly higher than that of *Monascus* M9 cultured in dark conditions (288 μg/g). The yield of citrinin decreased (272 μg/g) after 60 min of blue light irradiation per day, however the difference was not significant. Short-term blue light irradiation can stimulate *Monascus* to produce more citrinin, however long-time blue light irradiation reduces citrinin production. That is, the effect of blue light on citrinin production does not increase with the extension of illumination time, which indicates that blue light cannot directly regulate the production of citrinin. It may be that the blue light signal regulates citrinin production through other regulatory modes and that the light exerts a protective mechanism on *Monascus*.

### 2.2. Analysis of lncRNA in the Transcriptome of Monascus M9

After bioinformatics analysis, 1455 nucleotide sequences that conformed to the biological characteristics of lncRNA were screened from the transcriptome of monascus M9. Table 1 shows the results.

Some lncRNAs are involved in the basic processes of gene regulation, including the modification and structure of chromatin and direct transcriptional regulation. Gene regulation might occur in the form of cis or trans regulation. Cis regulation usually refers to the mode of action of DNA sequences from the same chromosome in the direct regulation of the expression of other nearby genes. In this study, we used the predicted genome annotation information of lncRNA and the reference genome information of the species to identify possible cis-acting target genes of lncRNA. LncRNA, which is normally transcribed in the same direction as the target gene in the promoter region, usually promotes the expression of the target gene, while reverse transcription inhibits the expression of the target gene. In this work, we analyzed the correlation between each lncRNA and the expression of mRNA, and we selected the corresponding relationship between lncRNA and mRNA with a correlation greater than or equal to 0.7 and P value less than or equal to 0.05 was selected for further study.

**Figure 2:**
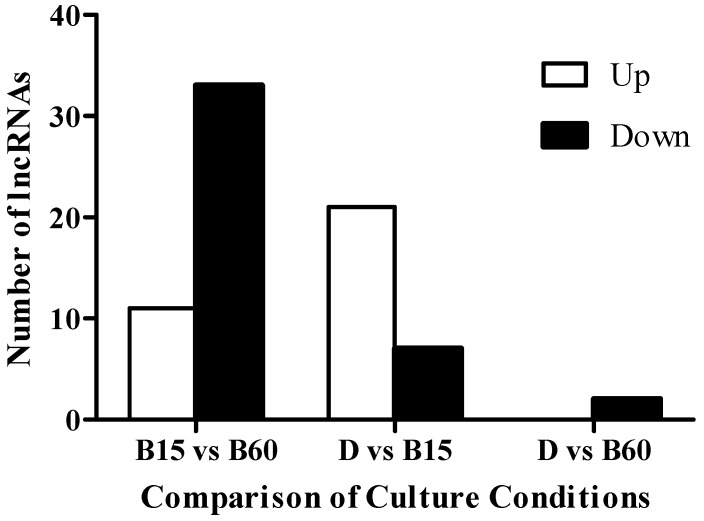
Numbers of lncRNAs with significant changes in expression under different blue light illumination conditions after eight days of culturing. The *Monascus* M9 culture was divided into three groups: dark culture conditions (D), blue light illumination for 15 min (B15), and blue light illumination for 60 min (B60) in one day; groups D, B15 and B60 had two parallels. Blue light was generated by a blue-LED box, which provided stable and uniform pure blue light (460 nm). The light intensity was 100 lux.

According to the criterion of difference significance (i.e., more than twice the difference in expression of lncRNA, and FDR ≤ 0.05), we screened and counted the up- and down-regulation of a significant difference in expression of lncRNA under blue light illumination. Figure 2 shows the results.

**Figure 3:**
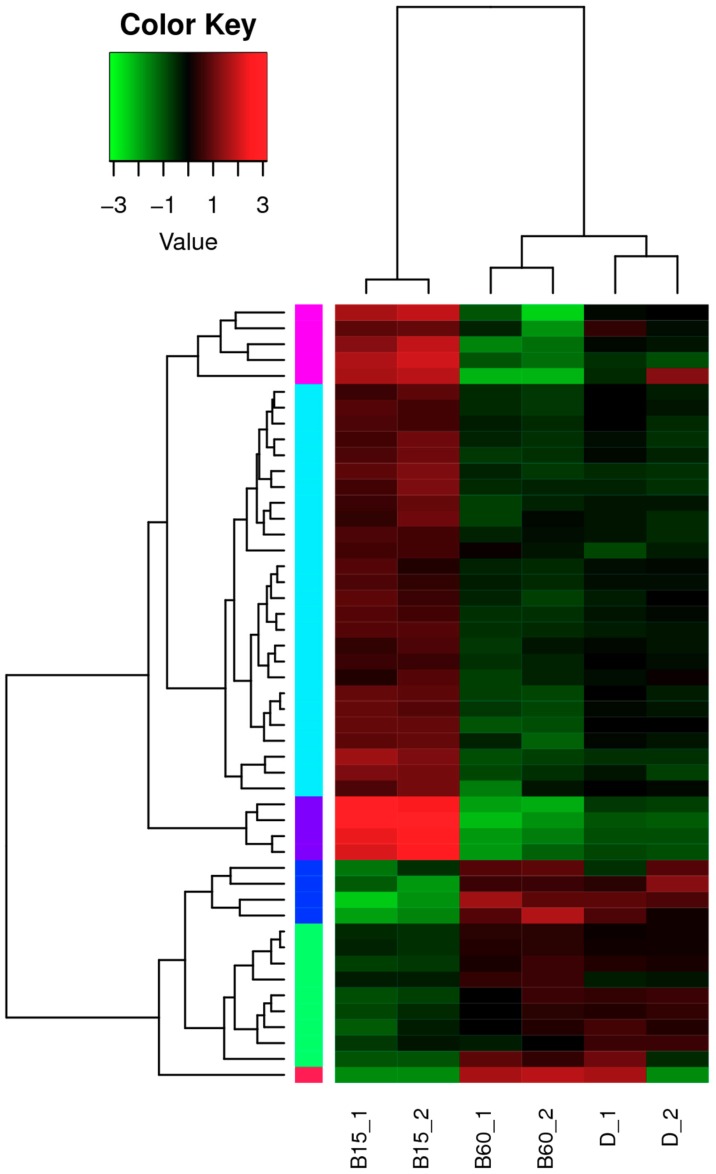
Cluster maps of differentially expressed long non-coding RNA (lncRNA) among all samples (false discovery rate (FDR) ≤ 0.05, fold change ≥ 2). The darker the color, the higher the amount of expression; each of the different color blocks on the left represents a class of transcript clusters with similar expression levels. Clustering was performed with log2 Reads Per Kilobase per Million mapped reads (RPKM) values, with red indicating high expression of transcript and green indicating low expression of transcript. Color variation from green to red indicates incrementally higher amounts of lncRNA expression. B15: illuminated with blue light for 15 min per day; B60: illuminated with blue light for 60 min per day; D: cultured in dark conditions. Two parallel samples were performed for each group. The blue light was generated by a blue-LED box, which provided stable and uniform pure blue light (460 nm). The light intensity was 100 lux.

Additionally, hierarchical clustering was used to analyze differentially expressed lncRNAs. Figure 3 shows the results.

All of the differentially expressed lncRNAs and mRNAs were analyzed, and a target mRNA (g4769.t1) named *mraox* was identified, which corresponded to the negative regulation of an lncRNA (TCONS_00015111), named *AOANCR*. LncRNA *AOANCR* is 398 bp in length, is located in the *mraox* promoter region, has a nucleotide similarity of 79% in the range of 389 bases to 894 bases of *mraox*, and it has reverse transcription with *mraox*. Based on the characteristics and sequence similarity of lncRNA and *mraox*, we speculate that lncRNA *AOANCR* is a reverse-regulated lncRNA of *mraox*.

After blast analysis, *mraox* and *Monascus ruber* M7 alternative oxidase gene (GenBank: FJ640864.1) reached 100% similarity, and *mraox* was therefore presumed to be an alternate oxidase-encoding gene. Studies have shown that the gene encoding alternative oxidase plays an important role in the signal transduction of environmental factors, such as pH and osmotic pressure. It is speculated that this gene might also be involved in light signal transduction and citrinin regulation.

### 2.3. Expression of lncRNA AOANCR and Mraox Detection in Different Blue Light Conditions

**Figure 4:**
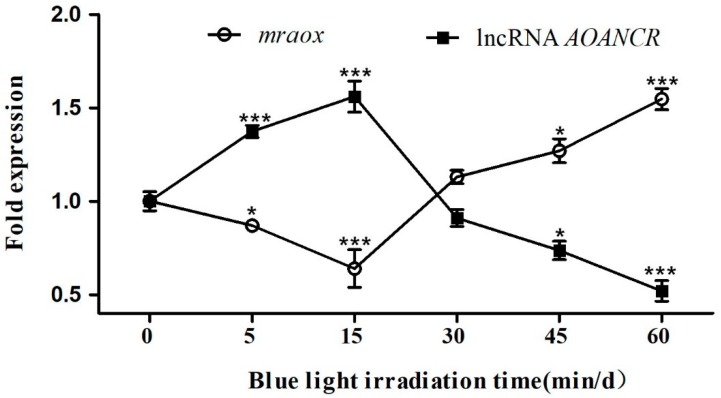
Expression of the *mraox* gene and lncRNA *AOANCR* under different blue light illumination conditions. The culture samples of *Monascus* M9 were irradiated with blue light for 0, 5, 15, 30, 45, and 60 min/d, and the cultures were sampled to detect gene expression by qPCR on the fourth day. Transcription levels were normalized to that of the *actin* gene. In order to standardize the results, we took the gene expression levels (*mraox* and lncRNA *AOANCR*) on the fourth day under dark conditions as the reference value (value 1). Data are expressed as the relative mRNA level for each gene and represent the average values of three separate experiments. The blue light was generated by a blue-LED box, which provided stable and uniform pure blue light (460 nm). The light intensity was 100 lux. Error bars represent the standard deviation (*n* = 3). *** *p* < 0.001, * *p* < 0.05 compared with gene ratio under dark conditions. T-test for the values was performed at *p* < 0.05.

In order to prove the expression relationship between lncRNA *AOANCR* and *mraox*, their expression was determined by qPCR. The expression of *mraox* and lncRNA *AOANCR* was detected in samples illuminated with blue light for 5 min., 15 min., 30 min., and 60 min. on the fourth day of cultivation. The expression of *mraox* and lncRNA *AOANCR* in a dark culture condition was taken as the reference value (value 1). The results are shown in Figure 4.

From the measured expression levels of *mraox* and lncRNA *AOANCR*, it can be seen that the trend was opposite for *mraox* and lncRNA *AOANCR*. In the case of blue light illumination for 15 min/d, the expression of lncRNA *AOANCR* was the highest, and the expression of *mraox* was the lowest. In the case of blue light illumination for 60 min/d, the expression of lncRNA *AOANCR* was the lowest and the expression of *mraox* was the highest. These opposite trends further prove that lncRNA negatively regulates *mraox*.

There is no linear relationship between gene expression and blue light illumination time, which might be related to the feedback inhibition of the blue light signal. Gene regulation by blue light is a hierarchical network regulation system, and different illumination times trigger different levels of the gene regulation network. In the case of *Neurospora crassa*, light-regulated genes can be divided into early response genes and late response genes [30]. WCC directly controls early light-regulated genes [31]. Early light-induced proteins regulate subsequent light-induced gene expression, while gene expression at the second level affects the next level. With the existence of photoadaptation, the expression of genes that was initially induced by light will be inhibited [32,33,34,35,36,37,38]. The downstream genes that were activated by the original light-regulated genes were subsequently suppressed, and the process of activation and suppression of multiple genes formed a hierarchical network of light regulation. The expression of lncRNA *AOANCR* and *mraox* determined in the present study suggests that photoadaptation also exists in *Monascus*.

### 2.4. Effects of Alternating Respiratory Pathway Mediated by Alternate Oxidase on Citrinin Synthesis in Monascus M9

**Figure 5:**
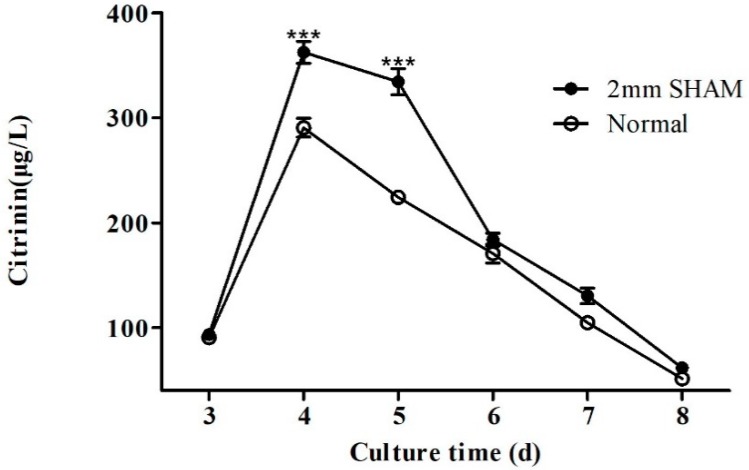
Effect of salicylhydroxamic acid (SHAM) on citrinin production. Citrinin content of *Monascus* M9 in YES broth assessed by HPLC. Normal means fermentation with normal YES medium. In the other group, 2 mm of SHAM was added to the YES medium before fermentation. Samples were taken from the same culture every 24 h from the third to the eighth day. Values are the averages of three independent experiments. Error bars represent the standard deviation (*n* = 3). *** *p* < 0.001 compared with citrinin production under normal conditions. T-test for the values was performed at *p* < 0.05.

From the aforementioned experimental results, it was found that blue light illumination can change the expression of lncRNA *AOANCR* and *mraox*, and the two genes have a reverse regulation relationship. However, fermentation studies are required to demonstrate whether the alternative respiratory pathway that is mediated by the *mraox* gene can regulate the synthesis of citrinin. 2 mm of the alternative oxidase pathway inhibitor salicylhydroxamic acid (SHAM) was added to the medium to detect the yield of citrinin after eight days of fermentation in order to study whether the alternative oxidation pathway affected the citrinin biosynthesis of *Monascus* M9. The experimental results are shown in Figure 5.

The results showed that the yield of citrinin produced in the normal medium (i.e., without the addition of SHAM) reached the highest value on the fourth day of culture, i.e., 290.5 μg/L. When SHAM was added to the culture, the citrinin yield also reached the highest value on the fourth day, i.e., 362.3 μg/L, a 24.7% increase as compared to the culture in the normal medium. When the alternation oxidase pathway was inhibited, the yield of citrinin was significantly increased. This result indicates that the alternative oxidase pathway can affect the production of citrinin. Blue light can regulate the expression of the *mraox* gene by regulating the expression of lncRNA *AOANCR*, which then regulates the alternate respiratory pathway, thereby affecting the production of citrinin.

### 2.5. Citric Acid and Aminophylline Regulate Citrinin Production by Regulating Mraox

After blue light illumination is applied to cells, cyclic adenosine monophosphate (cAMP) is formed in the cells, which in turn produces a series of downstream reactions [39]. Previous studies found that conidia fertilization of filamentous fungi cultured under dark conditions with the exogenous addition of cAMP was similar to that under blue light illumination. However, there is no similar phenomenon with the addition of exogenous cAMP if the photoreceptor gene is knocked out [40,41]. It can be seen that blue light is transmitted to the photoreceptor gene through cAMP and then regulates other physiological functions.

**Figure 6:**
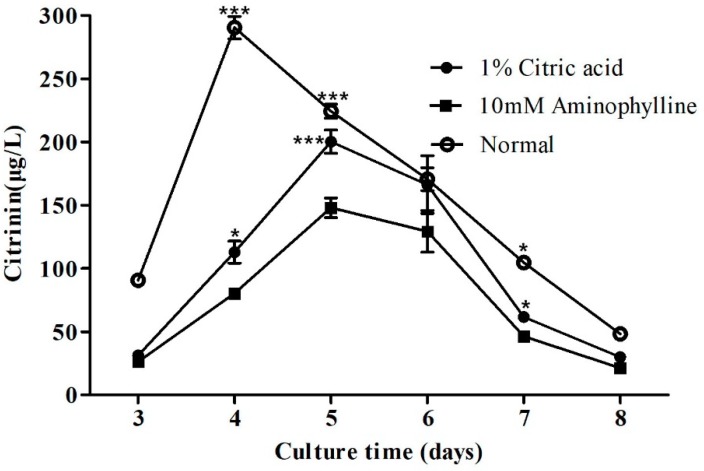
The effect of citric acid and aminophylline on citrinin production. The citrinin content of *Monascus* M9 in YES broth assessed by HPLC. Normal means fermentation with normal YES medium. In the other two groups, 1% citric acid and 10 mM aminophylline were added to the YES medium before fermentation, respectively. Samples were taken every 24 h from the third to the eighth day. Values are the averages of three independent experiments. Error bars represent the standard deviation (*n* = 3). *** *p* < 0.001, * *p* < 0.05 compared with citrinin production in normal conditions. T-test for the values was performed at *p* < 0.05.

The exogenous addition of citric acid and aminophylline can increase the cAMP content in fungal cells [42]. Citric acid and aminophylline were added to the medium in order to verify whether the regulatory effects of lncRNA *AOANCR* and *mraox* are also related to the cAMP pathway. The gene expressions of lncRNA *AOANCR* and *mraox*, and citrinin production, were detected. The experimental results are shown in Figure 6.

The results showed that citric acid and aminophylline can reduce citrinin production. In the culture in a normal medium, the maximum citrinin yield of 290.5 μg/L was produced on the fourth day. When citric acid or aminophylline were added to the culture medium, maximum citrinin yields of 200.3 and 148 μg/L, respectively, were produced on the fifth day, 31.3% and 26.1% lower than the yields under normal conditions, respectively.

**Figure 7:**
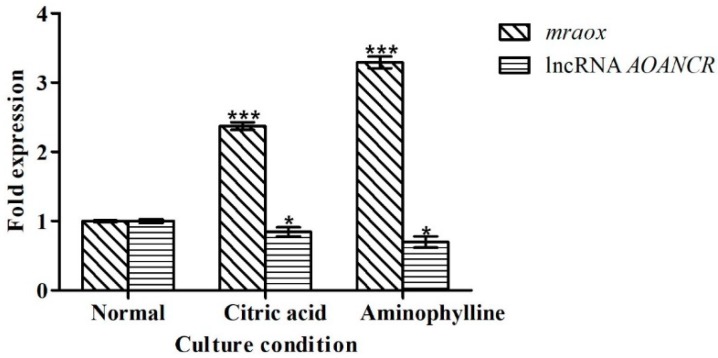
Expression of *mraox* and lncRNA *AOANCR* under different culture conditions. “normal” means fermentation with normal YES medium. In the other two groups, 1% citric acid and 10 mM aminophylline were added to the YES medium before fermentation, respectively. The cultures were sampled to detect gene expression by qPCR on the eighth day. Transcriptional levels were normalized to that of the *actin* gene. In order to standardize the results, we took the gene expression levels of *mraox* and lncRNA *AOANC* accumulated on the eighth day of samples cultured in normal medium as the reference value (value 1). Data are expressed as the relative mRNA level for each gene and they represent the average values from three separate experiments. Error bars represent the standard deviation (*n* = 3). *** *p* < 0.001, * *p* < 0.05 compared with the gene ratio of samples cultured in normal medium. T-test for the values was performed at *p* < 0.05.

Taking the gene expression in the normal medium as the reference value (value 1), the gene expression levels of *mraox* and lncRNA *AOANCR* were detected on the eighth day of culture. Figure 7 shows the results.

The results of the gene expression analysis showed that, after adding citric acid and aminophylline to the culture medium, the expression of *mraox* increased by 137% and 229%, respectively, and the expression of lncRNA *AOANCR* decreased by 15% and 30%, respectively. Citric acid and aminophylline both increased the expression of *mraox* and reduced the expression of lncRNA *AOANCR*.

Aminophylline and citric acid can stimulate the production of cAMP in cells. This suggests that blue light might also regulate the expression of lncRNA and *mraox* through the cAMP pathway. After aminophylline and citric acid were added to the culture medium, the expression of lncRNA and *mraox* showed opposite trends, which further confirmed the reverse regulation relationship between lncRNA *AOANCR* and *mraox*.

## 3. Discussion

As an environmental factor, blue light plays an important role in the synthesis of secondary metabolites of *Monascus*. Blue light signaling regulates the metabolism of *Monascus* in a complex regulatory relationship that involves multiple regulatory pathways [43]. Light can affect mycelial growth, promote the sporulation [21,22] of *Monascus*, and can also regulate the production of citrinin, monacolin K, and pigments, and the composition of pigments in gene expression level [23,24]. Di concluded that the effect of blue light on *Monascus* is global [28]. Blue light treatment can reduce the basal metabolic levels of *Monascus* M9, for example, in carbon and nitrogen metabolism, can degrade branched-chain amino acids, and can up-regulate aromatic amino acids. Blue light can also activate IP3/Ca^2+^ and DAG/PKC signaling pathways, inhibit the MAPK signaling pathway, and regulate the expression of key enzyme genes in metabolic pathways by regulating global transcription factors, thereby regulating the growth and secondary metabolism of *Monascus*. However, there are no reports of alternating respiratory pathways regulating citrinin production.

We found that the *mraox* gene is sensitive to blue light signals by bioinformatic analysis of transcriptome. We also found that the addition of the alternative respiratory pathway inhibitor SHAM to the medium can stimulate the production of citrinin, which indicates that the alternate respiratory pathway regulated by the *mraox* gene may be an important factor in regulating the production of citrinin. In fungi, alternate respiratory pathways exist in two forms [44], namely (1) alternative NADH dehydrogenase pathways, which are mediated by alternate NADH dehydrogenases, and (2) the alternate oxidase pathway, which is mediated by alternative oxidase [45]. The alternate oxidase pathway is the main pathway that links environmental factors and microbial metabolism. N stress, temperature, illumination, and high osmotic pressure [46,47,48,49] can affect the activity of alternative oxidase (AOX).

Blue light signals contribute to the production of reactive oxygen species (ROS) in organisms and increase photooxidative stress in cells [50], while intracellular ROS is a major factor in the activation of the alternate oxidase pathway [51]. On the one hand, AOX acts as an ROS scavenger, reducing ROS production [52]; on the other hand, AOX can inhibit excessive reduction of electron transport chain complexes in mitochondria, preventing excessive ROS production and activating the ROS clearance system to reduce ROS production [53,54]. Therefore, alternative oxidase is a protective mechanism that evolved to allow organisms to adapt to harsh environments. That is, organisms regulate their metabolism by alternative oxidase pathways to adapt to environmental changes.

The alternative oxidase pathway is an important pathway for *Monascus* to sense external environmental signals, especially environmental stress. The gene of the alternative oxidase, *mraox*, can enhance the resistance of *Monascus* to environmental stress and promote spore germination under environmental stress [30]. In this study, it was found that *mraox* is also one of the genes that regulate citrinin synthesis. It is generally believed that *aod-2* and *aod-5* are the regulatory genes of AOX [55]. In this study, we identified lncRNA *AOANCR*, whose base sequence is similar to the base sequence of the *mraox* gene and whose expression is opposite to that of the *mraox* gene; therefore, lncRNA *AOANCR* is presumed to be a negative regulator of *mraox*.

The regulation of lncRNA is a widely used approach in the medical field. For example, the feedback mechanism of the virus-induced lncRNA-mediated metabolic promotion of viral infection can be used as a potential target for developing broad-acting antiviral therapeutics [56]. Additionally, lncRNA is often associated with the growth and development of plants and microorganisms [57,58,59] and their adaptation to the surrounding environment [60]. Secondary metabolites are active substances secreted by fungi in harsh environments [61]. The external environment might regulate secondary metabolism through lncRNAs.

In *S. cerevisiae*, 11 lncRNAs are known to be related to metabolism, including galactose metabolism [62,63], phosphate metabolism [64], asparagine catabolism [65], fatty acid metabolism [66], biosynthesis of serine and glycine, etc. [67]. LncRNA plays an important role in the regulation of fungal metabolism. Unlike other modes of regulation, lncRNA-mediated regulation is more rapid and flexible, which is probably due to the fact that lncRNA can be quickly generated without the need for translation, and it rapidly degrades [68]. Therefore, the metabolism of citrinin can be regulated during fermentation while using lncRNA *AOANCR* as a target.

## 4. Conclusions

Citrinin is produced during fermentation, and it is therefore very important to regulate the metabolism of *Monascus* during fermentation to reduce the citrinin residue in red yeast products. In recent years, studies of the regulation of the metabolism of *Monascus* have focused on the effects of light with different intensities, different colors, and different wavelengths on sporulation and secondary metabolism [21,69,70]. Blue light can affect the expression of individual genes in the citrinin synthesis gene cluster of *Monascus*, however no studies have investigated the effect of blue light on lncRNA in *Monascus*. In this study, 1455 hypothetical lncRNAs were identified by bioinformatic analysis of the *Monascus purpureus* M9 transcriptome. The lncRNA *AOANCR* in the promoter region of the gene *mraox* has high homology and negative regulation. It is speculated that blue light may regulate lncRNA *AOANCR* through the cAMP pathway and then negatively-regulates *mraox* to effect citrinin production through the alternate respiratory pathway, which is a new mechanism for the regulation of citrinin synthesis. Further research is required to determine whether the metabolic regulation of environmental factors other than blue light also involves the participation of lncRNA *AOANCR*. In addition to light, other environmental factors, such as pH and temperature, can significantly affect the synthesis of citrinin [71,72]. LncRNA should also be considered as an important influencing factor in studies of the mechanism of the regulation of environmental factors to *Monascus*.

## 5. Materials and Methods 

### 5.1. Fungal Strain and Culture Conditions

*Monascus purpureus* M9 was maintained on potato dextrose agar (PDA) for five days at 30 °C in the State Key Laboratory of Food Nutrition and Safety, Tianjin, China. Spores were harvested with 3 mL sterile water and then inoculated into 75 mL seed medium (30 g rice powder/L, 2.5 g KH_2_PO_4_/L, 3 g NaNO_3_/L, and 1 g MgSO_4_·7H_2_O/L; the initial pH of the medium was adjusted to 4.5 with lactic acid) in 200 mL flasks. The cultures were incubated at 30 °C for 20 h with shaking at 180 rpm. For citrinin production and gene expression testing, 5 mL (10% *v*/*v*) of the seed fermentation broth was inoculated into 50 mL YES medium (normal medium, consisting of 40 g yeast extract/L and 160 g sucrose/L) in a 250 mL flask and incubated under static conditions at 30 °C [60,68].

### 5.2. Light Exposure Conditions

Light chambers were constructed and equipped with 9 W LEDs (460 nm) with a light intensity of 100 lux.

### 5.3. Transcriptome Data Acquisition

Transcriptome data were shared with Di [28]. The *Monascus* M9 culture was divided into three groups: dark culture condition, blue light culture condition for 15 min, and blue light culture condition for 60 min in one day, with two parallel groups.

### 5.4. Bioinformatics Analysis

LncRNA prediction: Firstly, using the latest genome annotation information that was provided by UCSC, Ensembl, and GENCODE, the transcripts overlapping with the known protein-coding, microRNA, Transfer RNA (tRNA), small nucleolar RNA (snoRNA), Ribosomal RNA (rRNA), and pseudogene annotation regions in the transcriptome were filtered. Based on the characteristics of lncRNA, transcripts containing only one exon, transcripts less than 200 bp in length, and transcripts that were supported by less than three reads, were filtered out. The HMMER-3 software [73] was used to evaluate all possible open reading frames of transcripts and filter out transcripts containing protein domains. The Coding Potential Calculator (CPC) software [74] was used to predict protein-coding potential and filter out the transcripts with protein-coding potential.

Cis-regulated lncRNA and trans-regulated lncRNA prediction: A region within 3000 bp upstream of the target gene was selected as the promoter region of the gene, and a cis-regulatory lncRNA was searched for in the promoter region of the gene. The analysis software was performed using the Bedtools intersect method. Trans-regulation is another way in which lncRNA acts on its target genes. The positional relationships between lncRNA and target genes were not considered, and base pairing directly identified lncRNA and target genes.

Blastn was used to identify the possible trans target genes for lncRNA. Gene difference analysis was carried out while using the DESeq2 software (v1.6.3) of the Bioconductor software package (v1.6.3, Bioconductor, Boston, MA, USA) [75]. The results of the DESeq detection were screened according to the difference significance criterion (i.e., differential lncRNA expression changed more than twice and FDR ≤ 0.05).

### 5.5. Analysis of Citrinin Production

Citrinin production was determined by high-performance liquid chromatography (HPLC) on a C18 column at 25 °C (5 μm, 250 mm × 4.6 mm) after filtration of the fermentation broth through a 0.45 μm filter. The mobile phase was acetonitrile/water/methanol (7:2:1, *v*/*v*) with the pH adjusted to 2.68 with H_3_PO_4_, running at 1 mL min^−1^. The element was monitored by fluorescence; the excitation and emission wavelengths were set at 331 and 500 nm, respectively. The standard Citrinin was used from Sigma.

### 5.6. QPCR Analysis

First-strand cDNA was synthesized while using the PrimeScript 1st Strand cDNA Synthesis Kit (TaKaRa, Japan), with the Oligo dT Primer 15. Gene expression was monitored by RT-qPCR and carried out using the SYBR Premix Ex Taq II (TaKaRa, Japan). RT-qPCR was performed while using the Stratagen Mx3000 P (Agilent, Santa Clara city, California, USA) with the following cycling program: hold at 95 °C for 30 s, followed by a three-step PCR (42 cycles of denaturation at 95 °C for 5 s, annealing at 60 °C for 30 s, and extension at 72 °C for 30 s) and dissociation curve analysis (at 95 °C for 15 s, annealing at 60 °C for 30 s, then collecting the dissociation curve from 60 °C to 95 °C, finally at 95 °C for 15 s). Table 2 shows the sequence of the primers used.

## Figures and Tables

**Table 1:** Biological characteristics of lncRNAs in M9.

Terms	Value
Total sequences	1455
Total bases	1056220
Min sequence length	202
Max sequence length	6927
Average sequence length	725.92
Median sequence length	310
As	25.29%
Ts	24.90%
Gs	24.78%
Cs	25.03%
(A+T)s	50.19%
(G+C)s	49.81%
Ns	0.00%

**Table 2:** Primers used in qPCR.

Primers	Sequence (5′–3′)
lncRNA *AOANCR*	
Forward primer	CGGGTGGCTAAGGGATTCAT
Reverse primer	GCCTGCTTGACTGCTGTTTC
*mraox*	
Forward primer	AAGAAACTGACCGACCGACC
Reverse primer	TGCAGTGTCCATTCCCCATC
*actin*	
Forward primer	GAGCGCGGCTACACCTTTAC
Reverse primer	GGGCAACGTAGCAGAGCTTCT
